# How Can Artificial Intelligence Make Medicine More Preemptive?

**DOI:** 10.2196/17211

**Published:** 2020-08-11

**Authors:** Usman Iqbal, Leo Anthony Celi, Yu-Chuan Jack Li

**Affiliations:** 1 Master Program in Global Health & Development PhD Program in Global Health & Health Security College of Public Health, Taipei Medical University Taipei Taiwan; 2 International Center for Health Information Technology Taipei Medical University Taipei Taiwan; 3 Laboratory for Computational Physiology Massachusetts Institute of Technology Cambridge, MA United States; 4 Division of Pulmonary, Critical Care and Sleep Medicine Beth Israel Deaconess Medical Center Boston, MA United States; 5 Department of Biostatistics Harvard TH Chan School of Public Health Harvard University Boston, MA United States; 6 Graduate Institute of Biomedical Informatics College of Medical Science and Technology Taipei Medical University Taipei Taiwan; 7 Department of Dermatology Taipei Municipal Wan-Fang Hospital Taipei Taiwan; 8 International Medical Informatics Association Geneva Switzerland

**Keywords:** artificial intelligence, digital health, eHealth, health care technology, medical innovations, health information technology, advanced care systems

## Abstract

In this paper we propose the idea that Artificial intelligence (AI) is ushering in a new era of “Earlier Medicine,” which is a predictive approach for disease prevention based on AI modeling and big data. The flourishing health care technological landscape is showing great potential—from diagnosis and prescription automation to the early detection of disease through efficient and cost-effective patient data screening tools that benefit from the predictive capabilities of AI. Monitoring the trajectories of both in- and outpatients has proven to be a task AI can perform to a reliable degree. Predictions can be a significant advantage to health care if they are accurate, prompt, and can be personalized and acted upon efficiently. This is where AI plays a crucial role in “Earlier Medicine” implementation.

In this paper we propose the idea that artificial intelligence (AI) is ushering in a new era of “Earlier Medicine.” Advanced digital health solutions play a significant role in improving health care by enabling accurate diagnosis, prescription automation, and early prediction of diseases with potential AI capabilities [1,2]. “Earlier Medicine” refers to a temporally predictive and proactive approach to individualized health enabled by innovative AI modeling plus longitudinal/personal health big data. It calls on medical practice to not just react or manage present situations but also medical events of the foreseeable future. Such an approach will save money, lives, and our health care ecosystem from inefficiencies and disintegration.

Monitoring the trajectories of both in- and outpatients has proven to be a task AI can perform reliably [3]. Predictive tools present a considerable advantage to medicine if they are accurate, prompt, customizable, and actionable. This is where AI plays a crucial role in “Earlier Medicine” implementation. In the late 1940s, Leavell and Clark [4] proposed three levels of prevention: primary, secondary, and tertiary. The term “primary prevention” was used to describe “measures applicable to a particular disease or group of diseases to intercept the causes of disease before they involve man” [4]. It is important to note, however, that the concept of prevention has evolved with time. Leavell and Clark's definition is disease-oriented, but the application of prevention overall extends beyond medical problems and addresses other societal concerns. Through prevention, we can create a society that fosters good health to improve the quality of care [5].

In the 1970s, Schwartz [6] speculated that by the year 2000, a sizeable amount of the thought process involved in medicine could be augmented by a division of AI known as expert systems. Recently, Topol [7] proposed that new technologies will improve the precision and accuracy of diagnosis and in doing so will enhance treatment selection. In the last 2 years, the World Health Organization (WHO) has been reviewing evidence on digital technologies from consulting experts around the world in order to assemble recommendations on ways such tools can be used for maximum impact on health care. In 2018, governments unanimously adopted a World Health Assembly resolution, calling on the WHO to develop a global digital health strategy to support national efforts to achieve universal health coverage [8]. The United States Food and Drug Administration has been proposing ways to establish greater oversight over this rapidly evolving segment of AI products to regulate these systems whose performance constantly change based on exposure to new patient data in different clinical settings [9]. Keeping these initiatives in mind, the saying, “prevention is better than cure” can be realized in a way like never before due to the advent of AI for “Earlier Medicine.” This brings us to propose the following levels of prevention—Actionable, Accurate, Timely, and Individualized (Figure 1).

**Figure 1:**
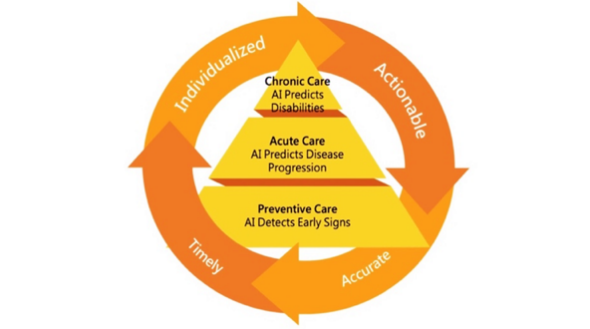
Artificial intelligence (AI) for "Earlier Medicine" with primary, secondary, and tertiary prevention levels.

First, we propose that primary prevention using AI should be targeted at those who are well. In the case of screening all healthy individuals for risk of disease, AI can be used for earlier rather than early detection for risk reduction. However, current screening protocols are oversimplified and suffer from low compliance. More robust models are needed, for example, for the risk prediction for nonmelanoma skin cancer [10]. Screening procedures are often the first step leading to early interventions that are more cost-effective than intervening once symptoms appear. In United States, positive results from screening mammograms were of little benefit; they resulted in increases to costs, and did little to nothing to ensure quality of life and decrease mortality rate [11]. Early detection serves little purpose for patients with an illness. We believe that society would benefit more from increased precision in the selection of groups for screening with AI-based earlier risk reduction using AI prediction technology. Other examples of the possible use of AI prediction technology is in the diagnosis of some forms of melanoma from an atypical mole such as cutaneous pigmented lesion screening, using smartphone‐generated images and clinical information simultaneously [12]. The incorporation of genomic information in electronic health records as part of one’s personalized treatment will drive the greater use of AI in primary prevention [13].

Secondly, we advocate that “Earlier Medicine” for secondary prevention is just as crucial. For a patient who is at risk of or suffering from a disease, AI can compute a management plan tailored to the patient’s individual needs. Secondary prevention AI includes recurrence prediction (eg, predicting non–ST-elevation myocardial infarction for patients with chest pain [14]).

Third, “Earlier Medicine” for tertiary prevention focuses on the deterrence of consequences of disease such as complications and disability. It also focuses on the overall improvement of quality of life through AI-based earlier interventions. Most countries are experiencing increases in the proportion of older people that comprise their population. It is estimated that by 2030, around 22% of the world’s population will be over 60 years of age [15]. There is an increasing prevalence of chronic disease in this age bracket globally and assisted daily living levels vary among regions [16,17]. Current health care systems that deal with this leave much to be desired in terms of long-term care, palliative care, and the expenses needed to maintain the system. Once a developed disease has been treated during its acute clinical phase, tertiary prevention seeks to soften the impact caused by the disease and potential complications on the patient's function, longevity, and quality of life. Tertiary prevention can include modifying risk factors. In cases where the condition is not reversible, tertiary prevention focuses on rehabilitation, assisting the patient to accommodate to disability [18]. For example, “Earlier Medicine” for tertiary prevention based on AI-computed individualized risk would help to prevent falls at home by 90% while reducing at least 10% of the dependent cost [19]. The key goal of tertiary prevention is to enhance quality of life by focusing on home health care services for either a short or long period as a result of illness, impaired health, old age, or other factors [20,21].

We earnestly believe that AI for “Earlier Medicine” will not only transform the practice of medicine but also radically reshape health care around the world. Harnessing the power of digital technologies is essential for achieving universal health coverage and to reviving humane medical practices for improving the quality of care [22]. The idea of prevention has evolved over the years from primary to tertiary and from a doctor-driven to patient-centered care model. AI for “Earlier Medicine” can serve as virtual medical assistants for clinicians, allowing for the resurgence of empathy-based care [23].

## Abbreviations

AI: artificial intelligence
WHO: World Health Organization
