# Correction: How Can Artificial Intelligence Make Medicine More Preemptive?

**DOI:** 10.2196/23645

**Published:** 2020-08-26

**Authors:** Usman Iqbal, Leo Anthony Celi, Yu-Chuan Jack Li

**Affiliations:** 1 Master Program in Global Health & Development PhD Program in Global Health & Health Security College of Public Health, Taipei Medical University Taipei Taiwan; 2 International Center for Health Information Technology Taipei Medical University Taipei Taiwan; 3 Laboratory for Computational Physiology Massachusetts Institute of Technology Cambridge, MA United States; 4 Division of Pulmonary, Critical Care and Sleep Medicine Beth Israel Deaconess Medical Center Boston, MA United States; 5 Department of Biostatistics Harvard TH Chan School of Public Health Harvard University Boston, MA United States; 6 Graduate Institute of Biomedical Informatics College of Medical Science and Technology Taipei Medical University Taipei Taiwan; 7 Department of Dermatology Taipei Municipal Wan-Fang Hospital Taipei Taiwan; 8 International Medical Informatics Association Geneva Switzerland

In “How Can Artificial Intelligence Make Medicine More Preemptive?” (J Med Internet Res 2020;22(8):e17211) the authors noted one error.

The first affiliation for author Usman Iqbal originally read:

College of Public Health, Taipei Medical University, Taipei, Taiwan

This was incomplete, and has been updated to read:

Master Program in Global Health & Development, PhD Program in Global Health & Health Security, College of Public Health, Taipei Medical University, Taipei, Taiwan

The correction will appear in the online version of the paper on the JMIR Publications website on August 26, 2020, together with the publication of this correction notice. Because this was made after submission to PubMed, PubMed Central, and other full-text repositories, the corrected article has also been resubmitted to those repositories

